# Millennial-Scale Temperature Change Velocity in the Continental Northern Neotropics

**DOI:** 10.1371/journal.pone.0081958

**Published:** 2013-12-02

**Authors:** Alexander Correa-Metrio, Mark Bush, Socorro Lozano-García, Susana Sosa-Nájera

**Affiliations:** 1 Instituto de Geología, Universidad Nacional Autónoma de México, Mexico City, Federal District, Mexico; 2 Department of Biological Sciences, Florida Institute of Technology, Melbourne, Florida, United States of America; University of Oxford, United Kingdom

## Abstract

Climate has been inherently linked to global diversity patterns, and yet no empirical data are available to put modern climate change into a millennial-scale context. High tropical species diversity has been linked to slow rates of climate change during the Quaternary, an assumption that lacks an empirical foundation. Thus, there is the need for quantifying the velocity at which the bioclimatic space changed during the Quaternary in the tropics. Here we present rates of climate change for the late Pleistocene and Holocene from Mexico and Guatemala. An extensive modern pollen survey and fossil pollen data from two long sedimentary records (30,000 and 86,000 years for highlands and lowlands, respectively) were used to estimate past temperatures. Derived temperature profiles show a parallel long-term trend and a similar cooling during the Last Glacial Maximum in the Guatemalan lowlands and the Mexican highlands. Temperature estimates and digital elevation models were used to calculate the velocity of isotherm displacement (temperature change velocity) for the time period contained in each record. Our analyses showed that temperature change velocities in Mesoamerica during the late Quaternary were at least four times slower than values reported for the last 50 years, but also at least twice as fast as those obtained from recent models. Our data demonstrate that, given extremely high temperature change velocities, species survival must have relied on either microrefugial populations or persistence of suppressed individuals. Contrary to the usual expectation of stable climates being associated with high diversity, our results suggest that Quaternary tropical diversity was probably maintained by centennial-scale oscillatory climatic variability that forestalled competitive exclusion. As humans have simplified modern landscapes, thereby removing potential microrefugia, and climate change is occurring monotonically at a very high velocity, extinction risk for tropical species is higher than at any time in the last 86,000 years.

## Introduction

As climates change, the capacity of species to migrate and accommodate to new configurations of their bioclimatic space is crucial to their survival. The current and future niche space for a species may be contiguous with their present range or it may require migration across an inhospitable landscape [1-3]. Fragmentation of populations and continuously changing occupiable niche space have been a feature of the Quaternary. Indeed, modern diversity patterns reflect bioclimatic envelopes, the dispersal capacity of species to reach potentially occupiable regions [4-6] and, eventually, species migration lags with respect to climate changes [7]. Climate change in the Quaternary may have contributed to speciation rates, but to date very limited evidence exists for extinction (none in Mesoamerica) [8]. Despite the hypothesized link between diversity and climate stability [9], little empirical information is available regarding climatic variability at fine-geographic and millennial-time scales in the Neotropics [10,11]. Furthermore, the lack of reliable quantitative estimators going beyond the historical record has impeded the documentation of the velocities at which climates changed in space and time. 

Available millennial-scale temperature migration velocities and the associated changes in the bioclimatic space have been exclusively based on models [5,9]. Consequently, the role of climatic dynamics in tropical diversity patterns and the threats that ecosystems face under modern climate change remain somehow speculative. Despite the lack of empirical data, basic predictions have emerged regarding the relationships between climate changes and diversity patterns: i) that migration rates need to be higher in large uniform landscapes than in steep heterogeneous ones [12,13], ii) that endemicity is high where climate migration rates are low [9], and iii) that forested landscapes have lower migration velocities than deserts and grasslands [12,14]. A disconnect exists, however, between possible rates, apparent rates, and modeled rates of migration of both species and climate [1,15]. 

**Figure 1:**
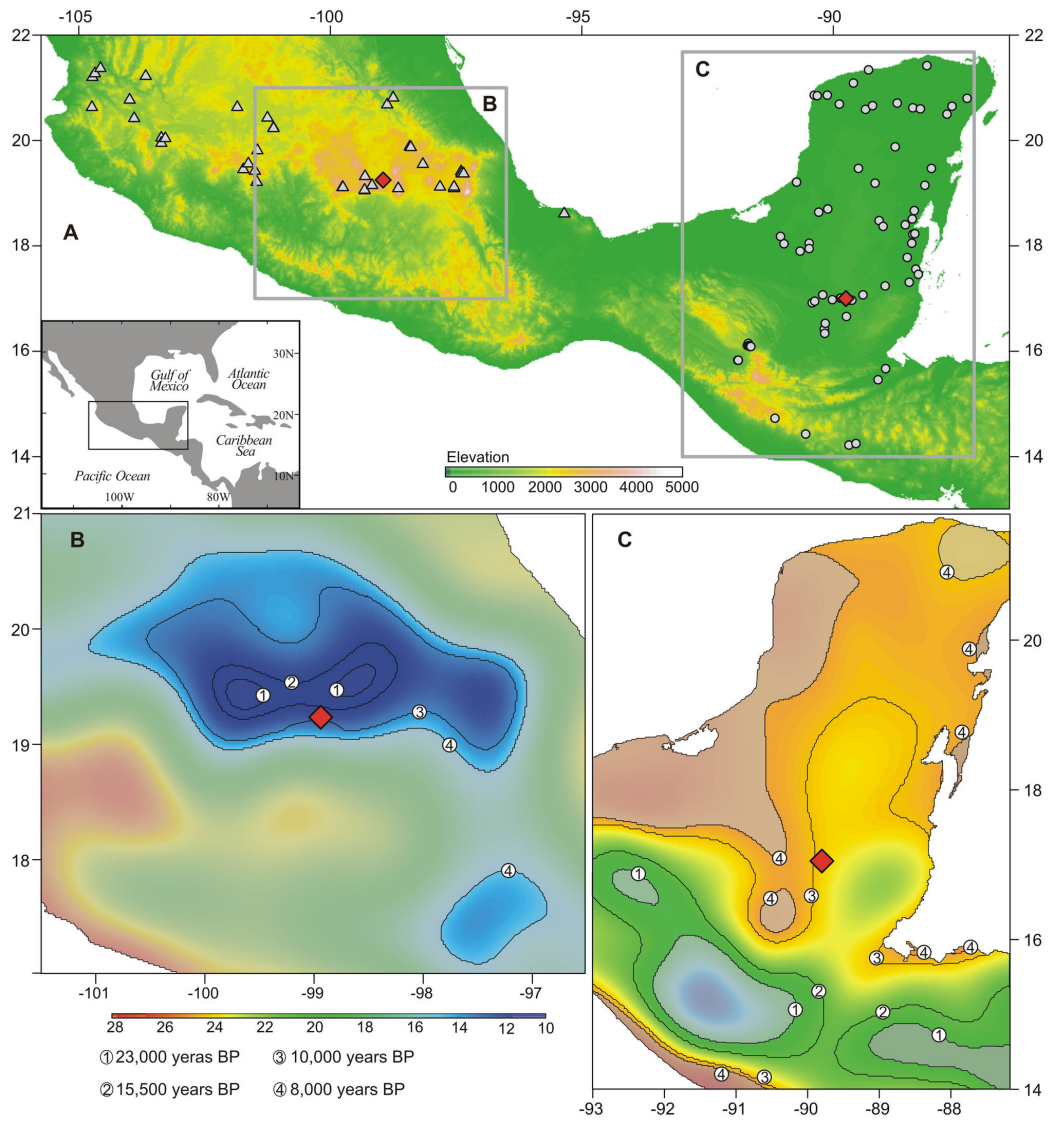
Study area. A. Fossil record sites (red diamonds) in an elevation map of central Mexico and the Yucatan Peninsula. Modern lakes sampled for pollen-temperature transfer functions shown in triangles (samples used to fit transfer function for Chalco record) and circles (samples used to fit transfer function for Petén-Itzá record). Lake Chalco (red diamond in B) and Lake Petén-Itzá (red diamond in C) in a map of modern temperatures in °C. Lines represent modern isotherms of temperatures experienced at each site at different times of the past. Shaded areas represent modern distribution of temperatures that have not been experienced at the places where the fossil records were recovered at any time during the studied time intervals.

In terms of modern climate change, observations suggest that ongoing displacement of the climatic space is outstripping the capacity for species to migrate [16], even in steep landscapes (slopes of ~45°) such as the tropical Andes [17]. Thus, it is critical to produce empirical quantitative measurements of climate change velocity in the past to assess extinction risk in modern landscapes and put modern climatic processes into a millennial-scale context. Two sedimentary records (Figure 1A) from Lake Petén-Itzá, Guatemala (110 m elev., lowland semi-deciduous tropical forest, basal age of ~86,000 years BP) and Lake Chalco, Mexico (2200 m elev., montane forest, basal age of ~30,000 years), demonstrated strong vegetational responses to climate change [18,19]. Here we present temperature records of both sites constructed using pollen-climate transfer functions. The estimated sequences in conjunction with digital elevation models allowed the quantification of temperature change spatial velocity for the late Quaternary. Thus, our study provides empirical millennial-scale data as a basis for estimating the magnitude of modern climate change and the probable role that Quaternary climates have played in configuring modern tropical diversity.

## Materials and Methods

### Temperature anomaly reconstructions

Temperature anomaly profiles for Lakes Petén-Itzá and Chalco for the last 30 and 86 thousand years, respectively, were derived through the pollen-based Synthetic Assemblages technique [20]. Pollen-temperature transfer functions were calibrated using modern data derived from 114 lakes (Figure 1A), whose mud-water interface sediments were analyzed. Because of the biogeographic divide that exists between the two regions under study [21], independent pollen-temperature transfer functions were built for central Mexico and the Yucatan Peninsula, using 40 and 74 lakes, respectively. Modern and fossil sediment samples were treated with standard protocols [22] and pollen was identified at magnifications of x40 and x100. Identifications were based on reference collections, pollen atlases [23,24], and the searchable database of Neotropical pollen [25]. Pollen sums for both modern and fossil datasets were set at 200 grains excluding dominant taxa (Moraceae, *Pinus*, and *Quercus*) and aquatics. Thus, pollen counts were between 400 and 3000 grains, but percentages were calculated based on the pollen sum of 200. Whereas modern samples from Central Mexico and temperature reconstruction based on the fossil pollen from Lake Chalco are a novel contribution of this paper, modern samples from the Yucatan Peninsula and adjacent mountains and temperature reconstruction for Lake Petén-Itzá have been previously published [10,20].

Mean annual temperature at each sampled modern location was derived from the CRU TS 2.1 database [26] to associate pollen spectra with modern temperatures. The Synthetic Assemblages transfer function relies on modeling pollen assemblages for individual temperature values [20] (anomalies with respect to modern conditions at the locations of the fossil records for this study). Once the ideal abundance of each taxon used for the transfer function was modeled, each fossil sample was compared with all the constructed assemblages. Then, the fossil sample was associated to the temperature anomaly that corresponded to the idealized synthetic assemblage showing the lowest dissimilarity (measured through Canberra distance). 

Whereas the reconstructed temperature anomalies for the Petén-Itzá record were based on a single fossil pollen sequence (PI-6, site 6 of Lake Petén-Itzá Drilling Project) [10], the reconstruction for Lake Chalco was based on three different sedimentary sequences (see Table S1 for chronologies): 103 samples from core Chalco B covering from ~3.5 to ~29.5 ka (all ages are expressed in thousands of calibrated years before present, ka), 112 from core Chalco D covering from ~3.5 to ~26 ka, and 145 samples from core Chalco E covering from ~1 to 20 ka. Whereas pollen records of Chalco cores B and D have been published elsewhere [19,27], the pollen sequence of core Chalco E is new to this study. Thus, a total of three sequences of temperature were built for the Chalco Basin, producing a total of 360 individual estimations (Figure S1). The unified temperature sequence was built by calculating the average reconstruction for each time point.

### Isotherm displacement velocities

The spatial distribution of temperatures at each time slice represented in the fossil record was estimated by re-draping a 30x30-m-resolution digital elevation model (DEM) derived from the STRM (Shuttle Radar Topography Mission) [28] with inferred temperatures. The re-draping was done using the reconstructed temperature for a time slice (T_r_) and the elevation of the coring site (E_r_) as a fixed point, and a moist-air adiabatic lapse rate of -5.5 °C per 1000 m [29]. Thus, temperatures for the other points of the DEM at the same time slice (T_i_) were derived through the expression T_i_ = T_r_ – 0.0055*(E_i_ – E_r_), where E_i_ are the elevations of each point of the DEM. Once the temperature raster was produced, it was summarized through a map of isotherms, making these generalizations available for each point in time that fossil pollen was available for (360 and 443 points for Chalco Basin and Lake Petén Itzá, respectively). Spatial climate change velocity was estimated using two time-consecutive isotherm maps M_1_ and M_2_, for time 1 and 2, respectively. The isotherm crossing the coring site in M_1_ was identified in M_2_, and then the minimum geographic distance among them was measured (See Figure S2 for an example with additional details). Then, such distance was divided by the time elapsed between M_1_ and M_2_, providing an estimation for the climate change velocity.

Past temperature sequences were randomly re-sampled 1000 times within the range produced by the estimated values ± error of the transfer functions to account for estimation error. Thus, 1000 sequences of temperature change velocity were produced for each location. Finally, temperature change velocity for each time slice was summarized through a probability density function that included all 1,000 estimates, assigning a probability density to each temperature change velocity.

## Results

**Figure 2:**
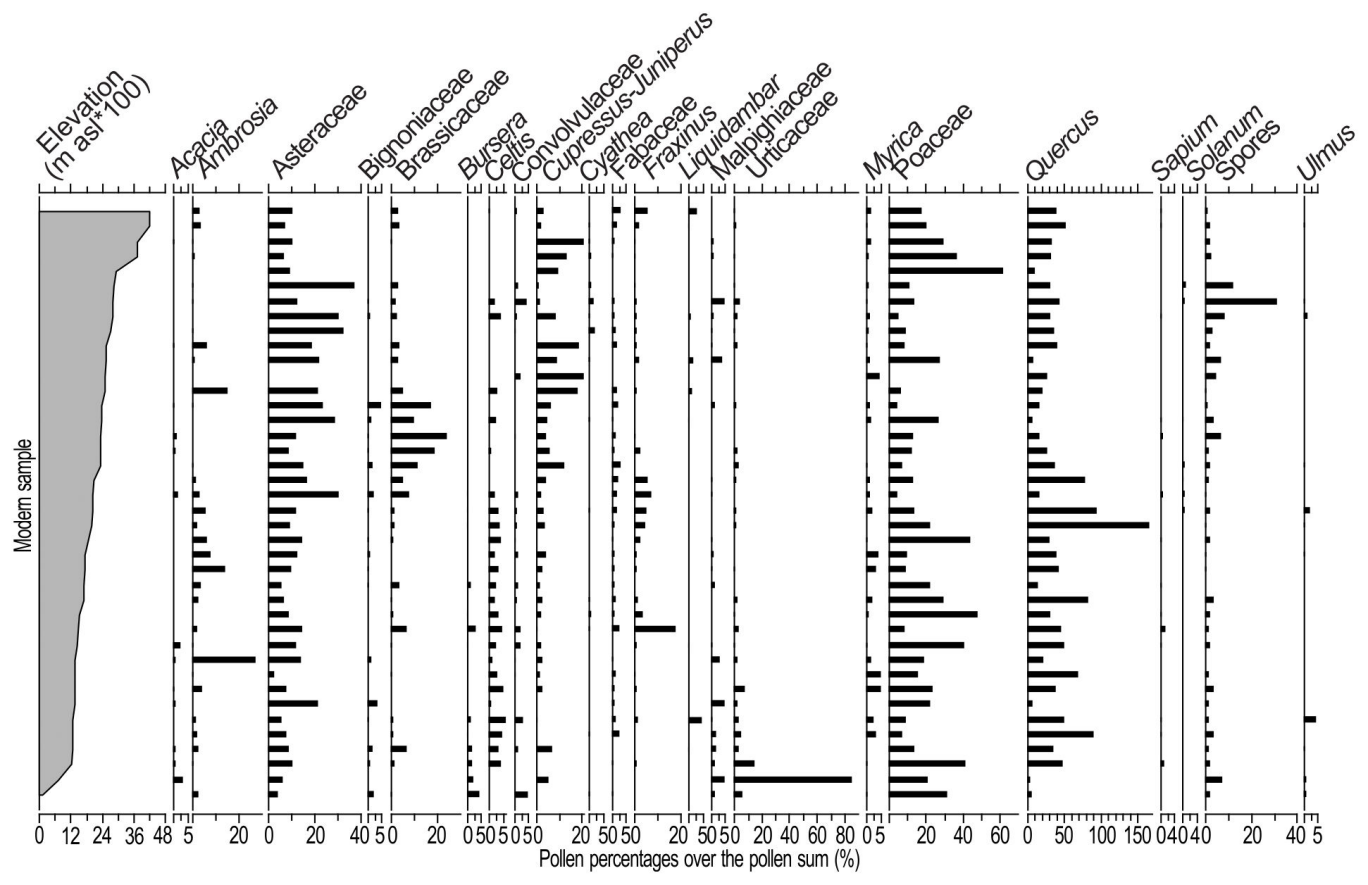
Percentages of selected taxa in modern mud-water interface samples from Central Mexico. Samples vertically organized according to elevation. Following the methodology used for climatic estimation of the record Petén-Itzá (for details see 10), percentages were calculated based on the pollen sum, which excluded *Quercus*, *Pinus*, and Moraceae/Urticaceae. Therefore, percentages of these taxa may sum to more than 100%.

**Figure 3:**
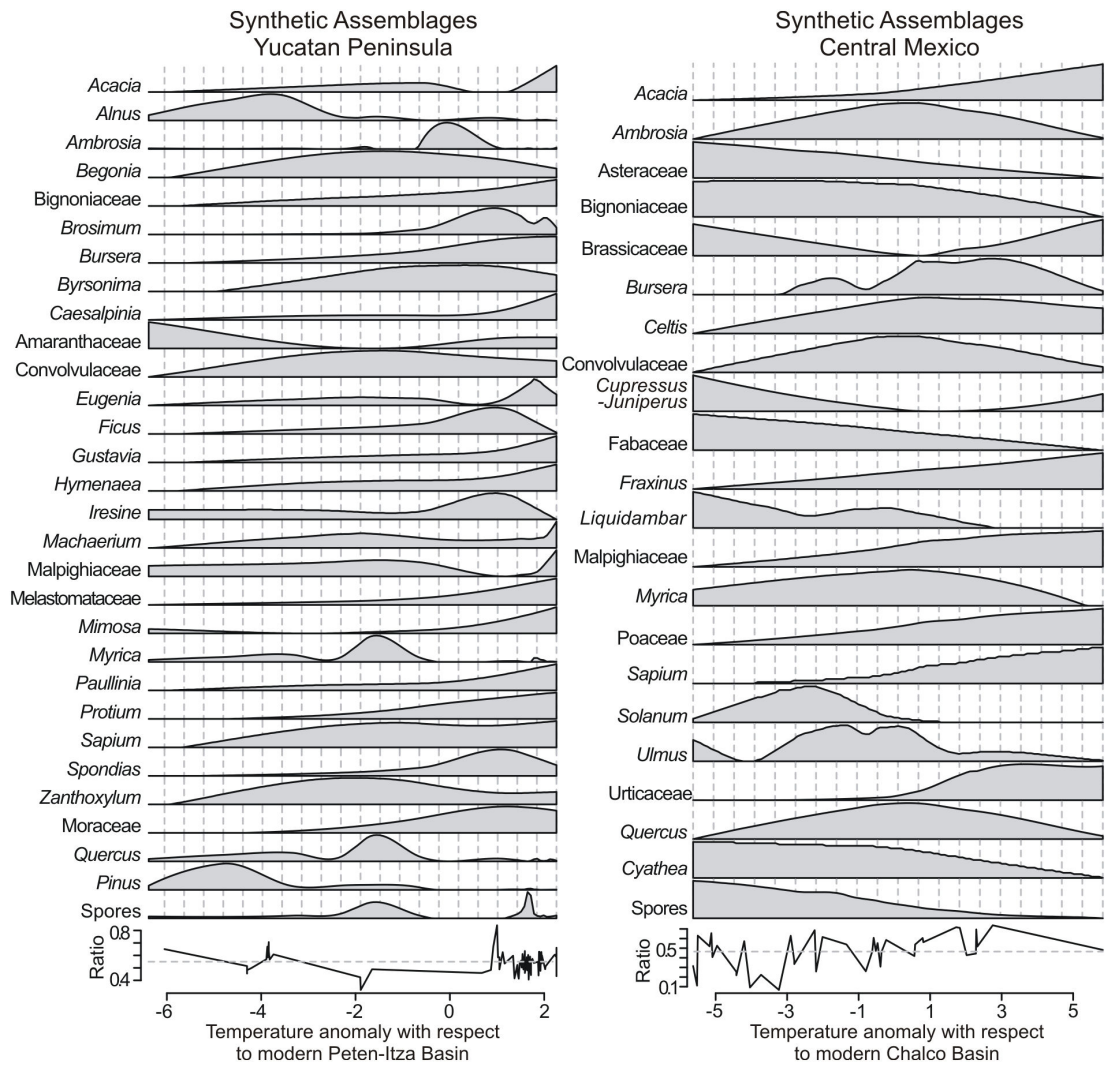
Synthetic pollen assemblages. Mean annual temperatures expressed in terms of the difference of each modern location with respect to Petén-Itzá Basin (left, modified from [10]) and Chalco Basin (right). Bottom panel represents the proportion of the total pollen dataset used to build the models that describe each taxon in terms of temperature. Abundances of taxa were scaled to a uniform scale for illustration purposes.

The optimal temperature transfer function for the Petén-Itzá area included 30 taxa [20], and the one for Chalco record included 22 taxa (Figures 2 and 3). Root of the mean squared error associated with the transfer functions were of 1.12 and 1.45 °C for Chalco and Petén-Itzá, respectively. The range of possible mean-annual-temperature anomalies contained in the Petén-Itzá transfer function was between -7.4 and +0.9 °C, and between -5.6 and +5.3 °C in the Chalco transfer function. This range was defined as the difference in mean annual temperature between the modern location of the coring sites and the sites where modern samples were collected.

**Figure 4:**
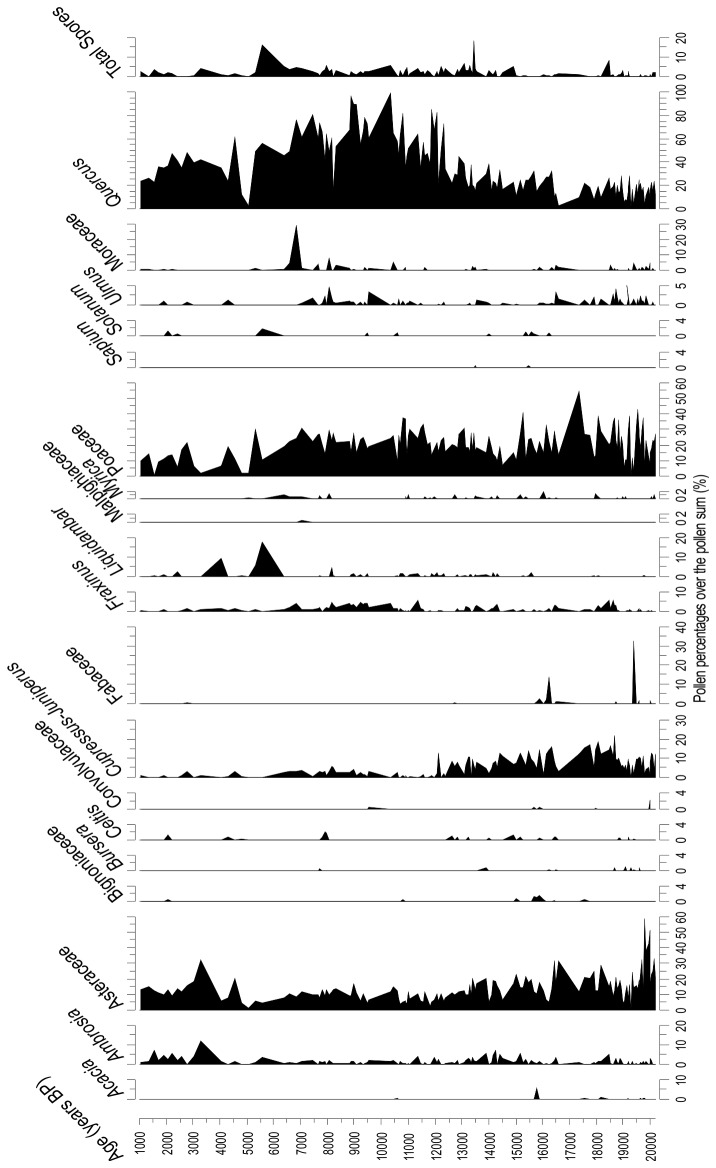
Percentages of selected taxa from core Chalco E. Following the methodology used for climatic estimation of the record Petén-Itzá (for details see 10), percentages were calculated based on the pollen sum, which excluded *Quercus*, *Pinus*, and Urticaceae. Therefore, percentages of these taxa may sum to more than 100%. Only taxa used for the pollen-climate transfer function are shown.

Pollen analyses of core Chalco E are new to this study (Figure 4) and show a pollen sequence that was consistent with the other Chalco records [19,27]. A transition from relatively dry open landscapes dominated by *Cupressus*-*Juniperus*, to a *Quercus* woodland, ~12.5 ka was evident. Peak Holocene warmth is shown in elevated levels of *Ulmus* and Urticaceae between ~9.5 and ~6 ka. From ~4 ka onward, human occupation is evidenced by high percentages of *Ambrosia* and Asteraceae, with the last 1000 years missing from the record probably due to filling for land reclamation associated with intensive human occupation.

**Figure 5:**
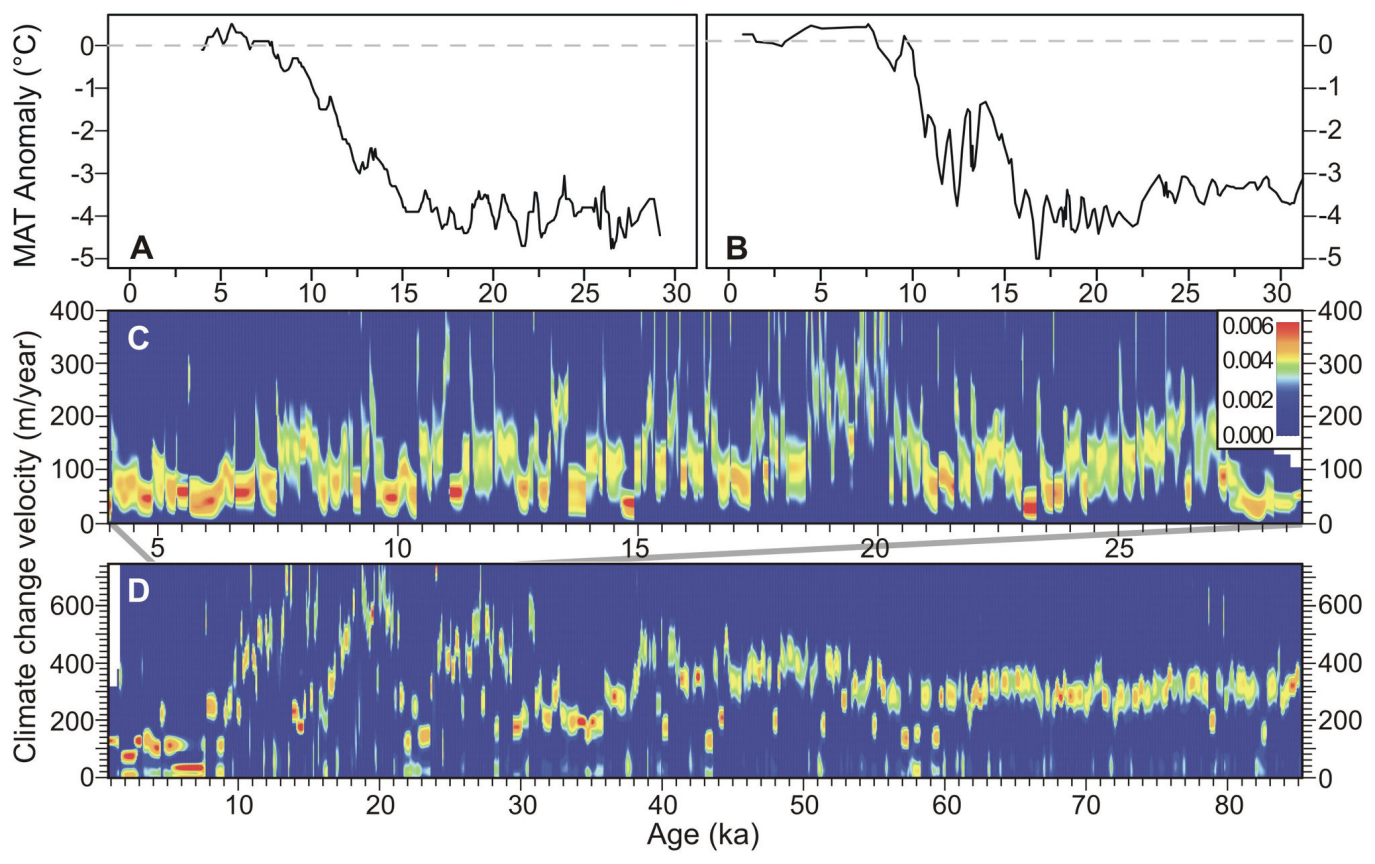
Mean annual air temperature anomaly reconstruction and temperature change velocity. Mean annual air temperature anomaly for (A) Chalco Basin and (B) Petén-Itzá, during the last 30,000 years; the entire 86,000-year-long series for Petén-Itzá can be viewed in Correa-Metrio et al. [10]. (C) Probability density of temperature change velocity for each sample point in the Chalco Basin during the last ~30,000 years. (D) Probability density of temperature change velocity for each sample point at Petén-Itzá during the last ~86,000 years. The probability density color code shown in panel C grades from red (maximum) to blue (minimum).

Average time between contiguous samples of the Chalco and Petén-Itzá reconstructions was 87 and 192 years, respectively. Reconstructed temperature profiles showed that during the last 30,000 years, the mean annual air temperature in Neotropical highlands and lowlands between 17 and 20° N was highly variable, but showed a parallel trend. Temperature anomaly estimations based on Lake Chalco’s fossil sequences showed a maximum cooling of ~4.5 °C during the Last Glacial Maximum, paralleling the results of the reconstruction of Petén-Itzá for the same time period [10] (Figures 5A and B).

Our results showed a scenario of realized temperature change velocities that, according to maximum probability densities, varied from 20 to 750 and from 30 to 400 m/yr for the Mesoamerican lowlands and highlands, respectively (Figure 5C and D). Patterns of temperature change velocity derived from both records showed important resemblances: a trend towards low velocities from ~27 to ~21 ka, high velocities from ~21 to ~18 ka (95% of the cases between 73 and 382, median 229, and between 343 and 742, median 584 m/yr, for Chalco and Petén-Itzá, respectively), highly variable velocities from ~18 to ~7.5 ka, and the lowest values from ~7.5 ka to present (95% of the cases between 32 and 155, median 45, and between 20 and 222, median 65 m/yr, for Chalco and Petén-Itzá, respectively). Going further into the past, the Petén-Itzá record showed temperature change velocities of around 300 m/yr before 60 ka. Between 60 and 38 ka, there was a increase of 80 m/yr in temperature change velocity and a noticeable increase in variability, ranging from 120 to 480 m/yr. The period between ~38 and 29 ka was more stable and characterized by velocities from 170 to 250 m/yr.

## Discussion

### Temperature anomaly reconstructions

Pollen-based temperature reconstruction from the sedimentary sequences of Lakes Chalco and Petén-Itzá suggested that climate changes during the last 30,000 years were relatively uniform at a regional scale. However, for the Holocene, estimations for Lake Chalco showed a cooling that took place between 4 ka and the most recent part of the record (Figure S1). This apparent cooling probably resulted from pollen spectra of this time period being derived from intense human land use and deforestation of the area. In fact, it has been reported that intensive human activity that included agriculture started in central Mexico around 4 ka [19,27,30,31]. Vegetation clearance for diverse purposes led to increased percentages of *Ambrosia* and Asteraceae during this time period, which under natural conditions were associated with cold to extremely cold environments (Figures 2 and 3). Thus, temperature estimations younger than 4,000 years in Chalco Basin were not used for calculating climate change velocities because the apparent cooling was an artifact of anthropogenic vegetation changes.

Although separated by 2 km vertically and >1000 km horizontally, Lakes Chalco and Petén-Itzá showed a regional drop of approximately 5 °C in mean annual temperature at the LGM (Last Glacial Maximum, from 19 to 23 ka [32]) (Figures 5A and B). This finding was consistent with little or no change in the moist-air adiabatic lapse rate through time, validating one of the assumptions we used for calculating temperature change velocities. However, estimates of temperature anomalies based on other proxies during the LGM and the deglaciation show coolings of as much as 8 °C [33,34], i.e. 3 °C lower than those produced by our pollen-climate transfer functions. This discrepancy could be the result of different proxies being more closely associated with seasonal temperatures or proxies reflecting either water or air temperatures. Specifically, because of the way we calibrated the transfer functions (synthetic assemblages constructed with taxa as a function of mean annual temperature) and because of the very nature of fossil pollen data (reflects regional vegetation and is affected by time averaging) [35], it is reasonable to assume pollen-based reconstructions reflect mean multi-annual air temperature. Given their large size and the central location where cores were recovered, Lakes Petén-Itzá and Chalco are very likely to reflect regional processes with little effect of local vegetation. Thus, the temperature anomalies we derived with our transfer functions showed changes that were not associated with temperature seasonality, but only with mean temperature through the year. As a consequence, the existent differences between our estimates of past temperature anomalies and those produced by other studies do not undermine our results. In fact, if our reconstructions were underestimating past temperature, they would put our analyses and the derived climate change velocities in the context of a conservative scenario, providing further support to our conclusions. Furthermore, the climate change scenarios constructed here were even more conservative when considering our estimates only included mean annual air temperature, leaving aside precipitation and seasonality changes, which could be important consequences of climate change in the tropics [16,36,37]. 

### Isotherm displacement velocities

Given the use of probability density functions to express temperature change velocities, exact estimates are not presented. Instead, all possible velocities were assigned a probability density represented by the color ramp (Figures 5C and 5D). Consequently, those values associated with the highest densities were the most likely and, therefore, the ones we use to construct our discussion. 

Temperature change velocities for both the lowlands and highlands were about two orders of magnitude higher than those reported for the region based on interpolations from climate models [9]. This discrepancy was probably the result of model-based estimates assuming a linear temperature trend from the Last Glacial Maximum to present. Temperature anomaly reconstructions, along with data from other areas [38-40], showed that warming trends during the deglaciation were far from linear, implying especially high climate-change velocities during high-frequency climatic oscillations. In fact, the highest velocities at both sites occurred between 20,000 and 10,000 years before present, the time that marked the end of the last ice age and was characterized by climatic instability [32]. Isotherm displacement velocity estimates could be affected by the temporal resolution of the data series, with highly resolved sections producing high temperature change velocities. However, the minimum estimates though the entire data series, 20 and 30 m/yr for the lowlands and highlands respectively, are still higher than those yielded by models [9]. Conclusively, the estimates for isotherm-displacement velocity we constructed offer a conservative perspective of late Quaternary climate change as a reliable context for modern climate change.

Temperature change velocities in the lowlands (Petén-Itzá) were almost twice as high as those of the highlands, highlighting the role of topography at modulating climate change-migration interactions. Whereas a change of 1 °C in the steep highlands implies a migratory distance of a few hundred horizontal meters, in the flat lowlands this would be associated with tens or even hundreds of kilometers [3] (Figures 1B and 1C). Paradoxically, despite the fact that highlands experience climate change velocities much lower than lowlands, extinction risks are concentrated in the former given the net loss of habitat associated with the upward migration of the isotherms and the conical geometry of mountains [3,13,41].

### Biogeographical implications

Reported migration velocities for trees based on modern and fossil data varied between 5 m/yr in densely forested Andean mountainsides [17] to 90 m/yr over flat terrain in North America [15]. These rates were clearly exceeded by our estimated temperature change velocities of 400-700 m /yr for the late Quaternary. Comparison of these rates leads to an expectation of a massive extinction of tree species during the late Quaternary in the Americas. However, extinction of plants during the late Quaternary has been low, with only one tree species known to have gone extinct in North America [42] and a second one suggested in the Andes [43]. Similarly, in central Mexico only one genus (*Picea*) showed regional extinction during this time period [44]. This apparent inconsistency calls for alternative scenarios regarding the role of migration as a linking mechanism between climate change and Neotropical patterns of diversity and endemism. Plant species survival during times of abrupt changes and unfavorable climates was probably mediated by microrefugia [13,15,45,46]. 

Microrefugia were environmentally anomalous locations that allowed a population to persist while the surrounding area would not have supported them [46]. Hence, despite an overall climate trend, a given species might still incongruously be present in the pollen spectra, and its probability of occurrence would not depend on regional environmental conditions. Additional mechanisms that could have promoted taxon persistence during times of abrupt climate changes or unfavorable conditions could be phenological and phenotypic plasticity of some species [47,48]. The most important aspects of microrefugia were that they reduced required migration rates, and provided increased genetic diversity [49]. Reid’s paradox [50] observed the required migration rates associated with repopulating Europe with trees following the last ice age were much higher than observed rates of spread. A red-tailed distribution where an extreme (low probability) dispersal event becomes of great significance is one way to overcome Reid’s Paradox [51]. Not all taxa, however, are suited to bursts of long-distance dispersal, and the presence of microrefugia offers an alternate explanation for overly-high migration rates [15,52]. Another significant effect of taxon isolation in a microrefugium would be the possibility of the expanding population being genetically distinct from the ‘mainland’ or ancestral population [15,49]. 

## Conclusions

During the last 30,000 years, climates of northern Central America were much more variable than suggested by model-based estimates [9]. The magnitude of local climatic changes had differential effects, mainly mediated by the topographic context in which they took place. Thus, mountainous areas with steep environmental gradients in central Mexico experienced relatively low climate change velocities, even under abrupt temperature oscillations. Additionally, the high topographic diversity of this landscape offered more potential for microrefugia, implying a higher potential for endemism than uniform landscapes in the lowlands. In fact, most continental areas identified by Sandel et al. [9] as having high amphibian, mammalian, and avian endemism coincided with mountain ranges, which were likely to provide such conditions. The exceptionally high endemism of tropical foothills was probably favored by highly variable climates that, together with disjunct populations that allowed rapid recolonization, forestalled competitive exclusion. This scenario offers an alternative view to the usual depiction of stable climate promoting high diversity [53,54].

Our results show that temperature change velocities in northern Central America were much higher than those produced by models, but significantly slower than those reported for the past 50 years [16] and those projected for the near future [3]. Continental temperature change velocity observed for the period between 1960 and 2009 for the northern hemisphere is about 3000 m/yr [16], a figure four times higher than the highest values we found for the late Quaternary. The capacity of species to disperse varies considerably [6,14,55], but under this scenario sessile organisms with long maturation phases, such as some tropical trees, may be unusually vulnerable to extinction given their low migration velocities [15,17]. Thus, conservation goals should prioritize areas with potential for rapid migration and high topographic diversity [3,13]. 

Microrefugia, environmental pockets that were not associated with regional environmental drivers, probably provided temporal means for species persistence through times of unfavorable conditions and posterior recolonization of the landscape. Climate change velocities measured for the last decades [16] are between four and seven times faster than the maximum velocities we calculated for the last 30,000 years, representing a record climatic pressure on terrestrial ecosystems. Our results emphasize the importance of habitat heterogeneity in the capacity of a diverse flora and fauna to survive abrupt climate change, although we are not questioning the interpretation of the paleoenvironmental records derived from the Neotropics. As humans have simplified modern landscapes and climate change is occurring monotonically at a very high velocity, extinction risk for tropical species is at an all-time high.

According to the high temperature change velocities and the low extinction rates, we suggest that disjunct population persistence played a major role at maintaining the diversity inherited from the Tertiary. Disjunct populations, which persisted either through microrefugia or suppressed individuals, were maintained over relatively short-time scales and were probably favored by high climatic variability. Had the climatic trend been more long-term, metapopulations would not have been able to persist, and competitive exclusion would have impoverished diversity [49]. Accordingly, centennial-scale oscillatory climatic variability probably plays a major role at maintaining high diversity in the tropics, whereas long-term monotonic trends favor extinction. In this context, and given the improbability that modern CO_2_ concentrations will return to their preindustrial state [37], modern global warming occurring at least four times faster than during the late Quaternary represents a major threat for tropical diversity. 

A distinction should be made between anthropogenic habitat fragmentation, which induces edge effects and microclimatic change [56] and does not replicate the existence of past (climatically stable) microrefugial fragments. Microrefugia had specific environmental attributes that allowed species to survive while the matrix around them changed. Contrastingly, fragmented populations isolated by development are merely the last ones standing, and actually have lower probabilities of stability than an average for the prior population[49]. During the past 8000 years humans have progressively altered landscapes through deforestation, burning and soil erosion [30,31,57]. A basic observation of the Anthropocene is that humans have simplified landscapes to suit their needs, thereby reducing heterogeneity. As plant migration is not fast enough and is hindered by a fragmented landscape, and microrefugia are eliminated from landscapes by millennia of development, the capacity for species’ survival is now at an all-time low. Many species will not be able to track the ongoing displacements of their bioclimatic space [12,58] and others will suffer important range losses in the high mountains [3,41], possibly leading to a massive extinction.

## Supporting Information

Figure S1 **Annual temperature anomaly reconstruction for Lake Chalco.** Punctual estimation using pollen samples from three different cores represented by dots (hollow circles core Chalco B; triangles core Chalco D; and diamonds core Chalco E). Mean composite reconstruction in blue line.
(JPG)

Figure S2 **Example of isotherms’ displacement and climate change velocity estimation.** Isotherms for 85,430 and 85,262 years BP, and temperature change velocity between them.
(JPG)

Table S1 **Radiocarbon ages of Lake Chalco cores calibrated according to Fairbanks et al 2005 [1].**
(PDF)
