# Supplementary figures and images for: Millennial-Scale Temperature Change Velocity in the Continental Northern Neotropics
Source: PLoS One. 2013 Dec 2;8(12):e81958. doi: 10.1371/journal.pone.0081958 (PMC3846729; doi:10.1371/journal.pone.0081958)

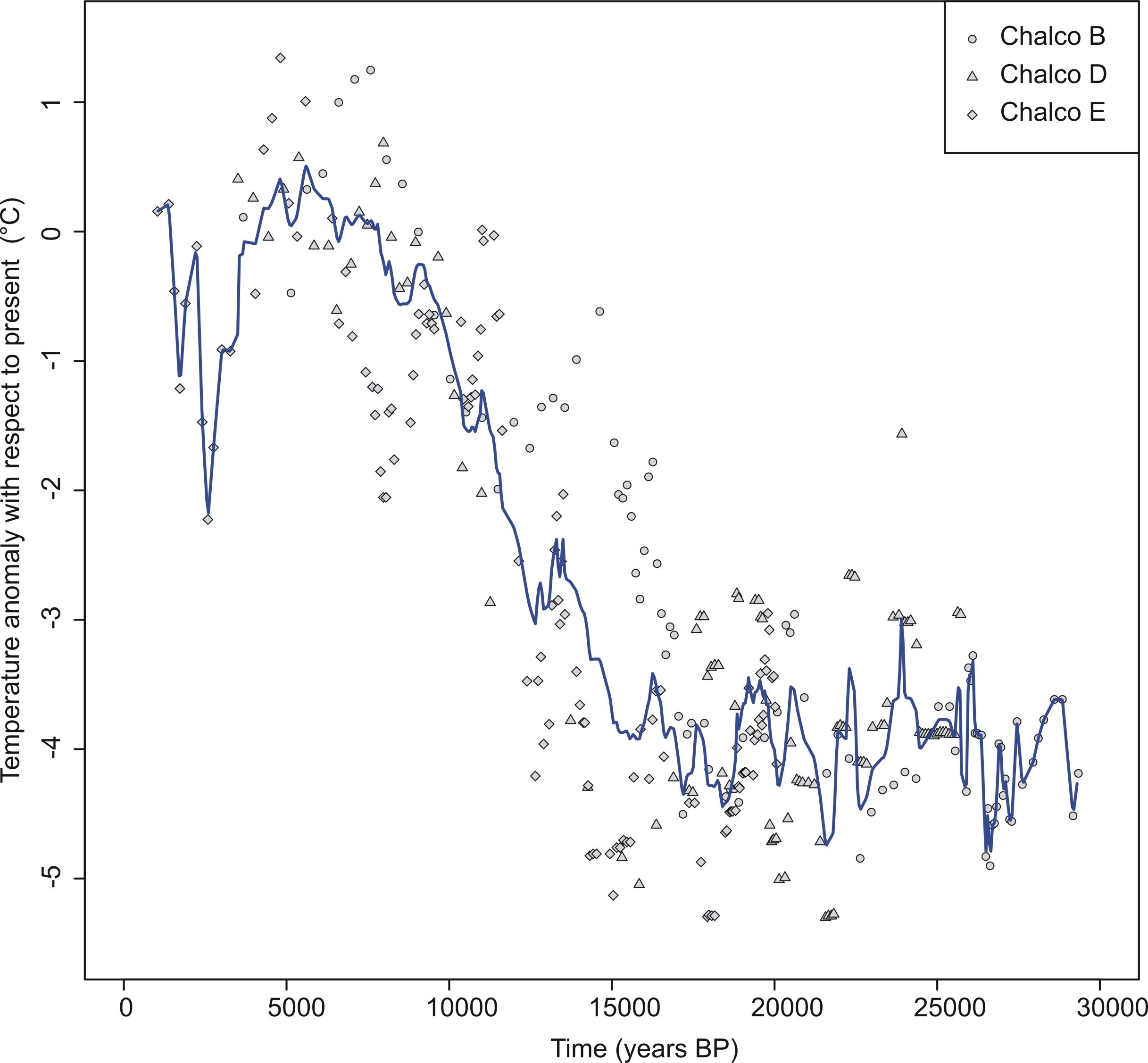

Supplement: Figure S1 — Annual temperature anomaly reconstruction for Lake Chalco. Punctual estimation using pollen samples from three different cores represented by dots (hollow circles core Chalco B; triangles core Chalco D; and diamonds core Chalco E). Mean composite reconstruction in blue line. (JPG) [file pone.0081958.s001.jpg]

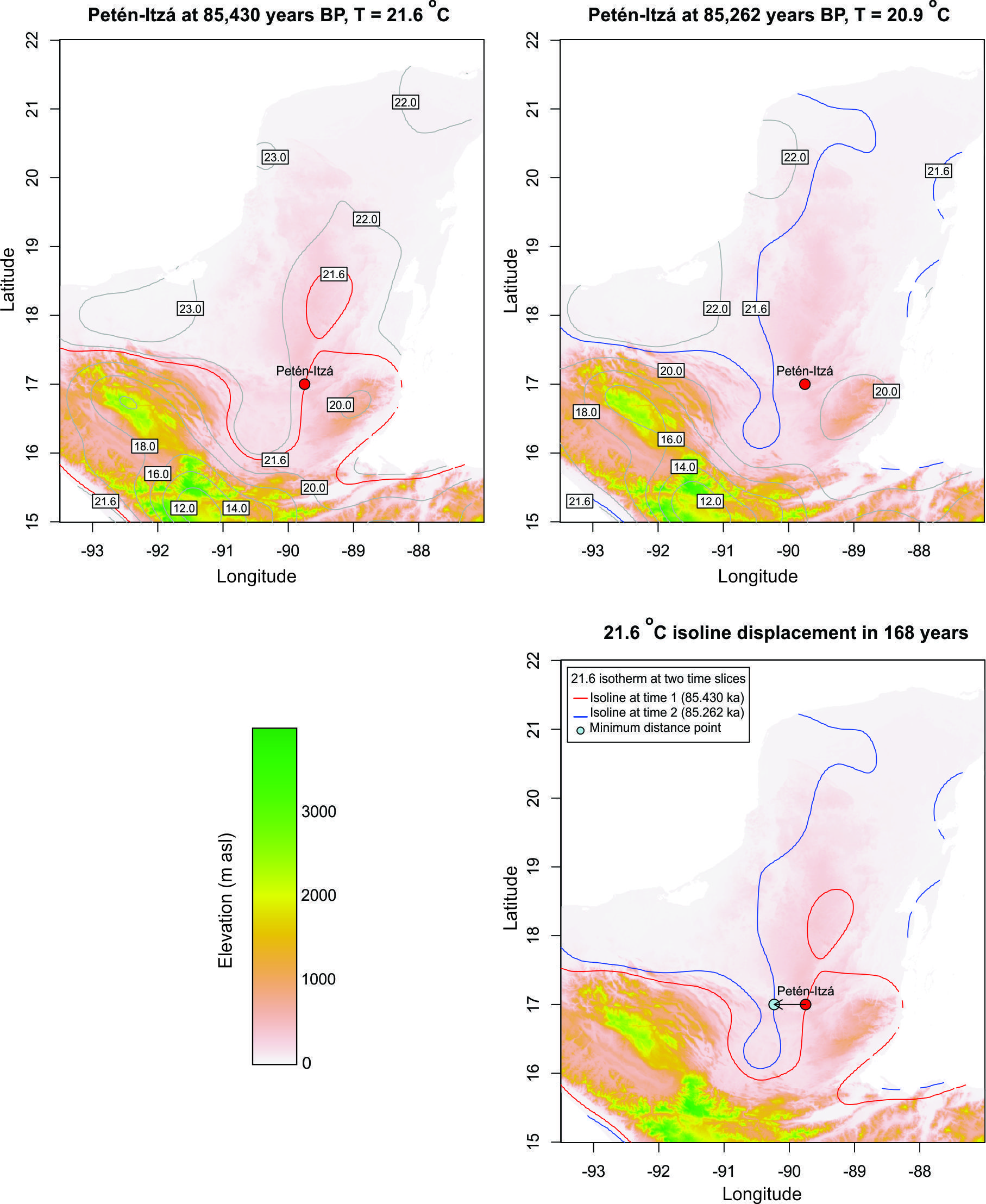

Supplement: Figure S2 — Example of isotherms’ displacement and climate change velocity estimation. Isotherms for 85,430 and 85,262 years BP, and temperature change velocity between them. (JPG) [file pone.0081958.s002.jpg]
