# Supplementary material for: Millennial-Scale Temperature Change Velocity in the Continental Northern Neotropics
Source: PLoS One. 2013 Dec 2;8(12):e81958. doi: 10.1371/journal.pone.0081958 (PMC3846729; doi:10.1371/journal.pone.0081958)
Supplement: Table S1 — Radiocarbon ages of Lake Chalco cores calibrated according to Fairbanks et al 2005 [1]. (PDF) [file pone.0081958.s003.pdf]

Supporting Information

Table S1. Radiocarbon ages of Lake Chalco cores calibrated according to Fairbanks et al 2005 [1].

Core	c^{14} Age	±	Depth (m)	Calibrated age (years BP)	±	Reference
Chalco B	10528	74	2,55	12476	101	[2]
Chalco B	12800	90	3,23	14914	133	[3]
Chalco B	17450	170	7,57	20640	210	[3]
Chalco B	21600	1050	9,10	25896	1322	[3]
Chalco B	22720	220	11,20	27290	282	[4]
Chalco B	26910	600	14,20	32208	659	[4]
Chalco D	5725	179	0,93	6522	198	[3]
Chalco D	5330	235	1,75	6012	257	[3]
Chalco D	9395	255	2,60	10648	360	[3]
Chalco D	12520	135	3,56	10504	248	[3]
Chalco D	14610	470	4,37	17365	788	[3]
Chalco D	16820	195	6,69	19977	229	[3]
Chalco D	19040	390	8,18	22668	466	[3]
Chalco E	2645	55	0,80	2756	35	[5]
Chalco E	6800	70	1,75	7640	54	[6]
Chalco E	8315	115	2,55	9314	151	[6]
Chalco E	9370	90	3,23	10589	126	*
Chalco E	9900	80	3,59	11300	105	*
Chalco E	9767	75	4,11	11192	65	[6]
Chalco E	11414	78	4,80	13264	99	[6]
Chalco E	12952	99	5,68	15088	147	[6]
Chalco E	12454	100	6,23	14385	206	*
Chalco E	14013	129	7,08	16360	216	[5]
Chalco E	13988	100	7,28	16321	180	[5]
Chalco E	14293	92	7,55	16792	171	[5]
Chalco E	14915	102	8,02	17934	247	*
Chalco E	15493	130	8,71	18712	126	[6]
Chalco E	15239	104	9,88	18478	141	*

* New to this study

Figure S1. Annual temperature anomaly reconstruction for Lake Chalco. Punctual estimation using pollen samples from three different cores represented by dots (hollow circles core Chalco B; triangles core Chalco D; and diamonds core Chalco E). Mean composite reconstruction in blue line.

Figure S2. Example of isotherms' displacement and climate change velocity estimation for two contiguous time slices at Petén-Itzá (89.75°W 17°N). All panels use modern elevation (m asl) as a reference (digital elevation models from the SRTM [7]). **A.** Isotherms for 85,430 years BP (M1), according to the temperature anomaly estimated based on the pollen data of the PI-6 record (-3.54 °C with respect to modern temperature). **B.** Isotherms for 85,262 years BP (M2), according to the temperature anomaly estimated based on the pollen data of the PI-6 record (-4.27 °C with respect to modern temperature). **C.** Isotherms of 21.6 °C at time 1 and 2; at time 1, temperature in Petén-Itzá was ~21.6 °C, and the nearest point to the isotherm associated this temperature at time 2 was 90.232°W 17°N, for a geographic distance of 51,544.48 m. Thus, the temperature change velocity associated with this time interval was calculated by dividing the isotherm displacement by the time ellapsed, resulting in 306.81 m/year.

References

1. Fairbanks RG, Mortlock RA, Chiu T-C, Cao L, Kaplan A, et al. (2005) Radiocarbon calibration curve spanning 0 to 50,000 years BP based on paired 230Th/ 234U/ 238U and 14C dates on pristine corals. Quaternary Science Reviews 24: 1781-1796.
2. Lozano-Garcia MS, Ortega-Guerrero B (1998) Late Quaternary environmental changes of the central part of the Basin of Mexico: correlation between Texcoco and Chalco basins. Review of Palaeobotany and Palynology 99: 77-93.
3. Lozano-Garcia MS, Ortega-Guerrero B, Caballero-Miranda M, Urrutia-Fucugauchi J (1993) Late Pleistocene and Holocene paleoenvironments of Chalco Lake, Central Mexico. Quaternary Research 40: 332-342.
4. Caballero-Miranda M, Ortega-Guerrero B (1998) Lake levels sice about 40,000 years ago at Lake Chalco, near Mexico City. Quaternary Research 50: 69-79.

5. Ortega-Guerrero B, Newton AJ (1998) Geochemical characterization of Late Pleistocene and Holocene tephra layers from the Basin of Mexico, Central Mexico. Quaternary Research 50: 90-106.
6. Ortega-Guerrero B, Thompson R, Urrutia-Fucugauchi J (2000) Magnetic properties of lake sediments from Lake Chalco, central Mexico, and their palaeoenvironmental implications. Journal of Quaternary Science 15: 127-140.
7. Farr TG, Rosen PA, Caro E, Crippen R, Duren R, et al. (2007) The Shuttle Radar Topography Mission. Reviews of Geophysics 45: RG2004.
